# Supplementary material for: Integrative RNA-Seq and H3 Trimethylation ChIP-Seq Analysis of Human Lung Cancer Cells Isolated by Laser-Microdissection
Source: Cancers (Basel). 2021 Apr 5;13(7):1719. doi: 10.3390/cancers13071719 (PMC8038546; doi:10.3390/cancers13071719)
Supplement: Supplementary file 1 [file cancers-13-01719-s001.zip › Supplementary Figures.docx]

**Supplementary Material: Integrative RNA-Seq and H3 Trimethylation ChIP-Seq Analysis of Human Lung Cancer Cells Isolated by Laser-Microdissection**

**Quang Ong, Shingo Sakashita, Emi Hanawa, Naomi Kaneko, Masayuki Noguchi and Masafumi Muratani**

| 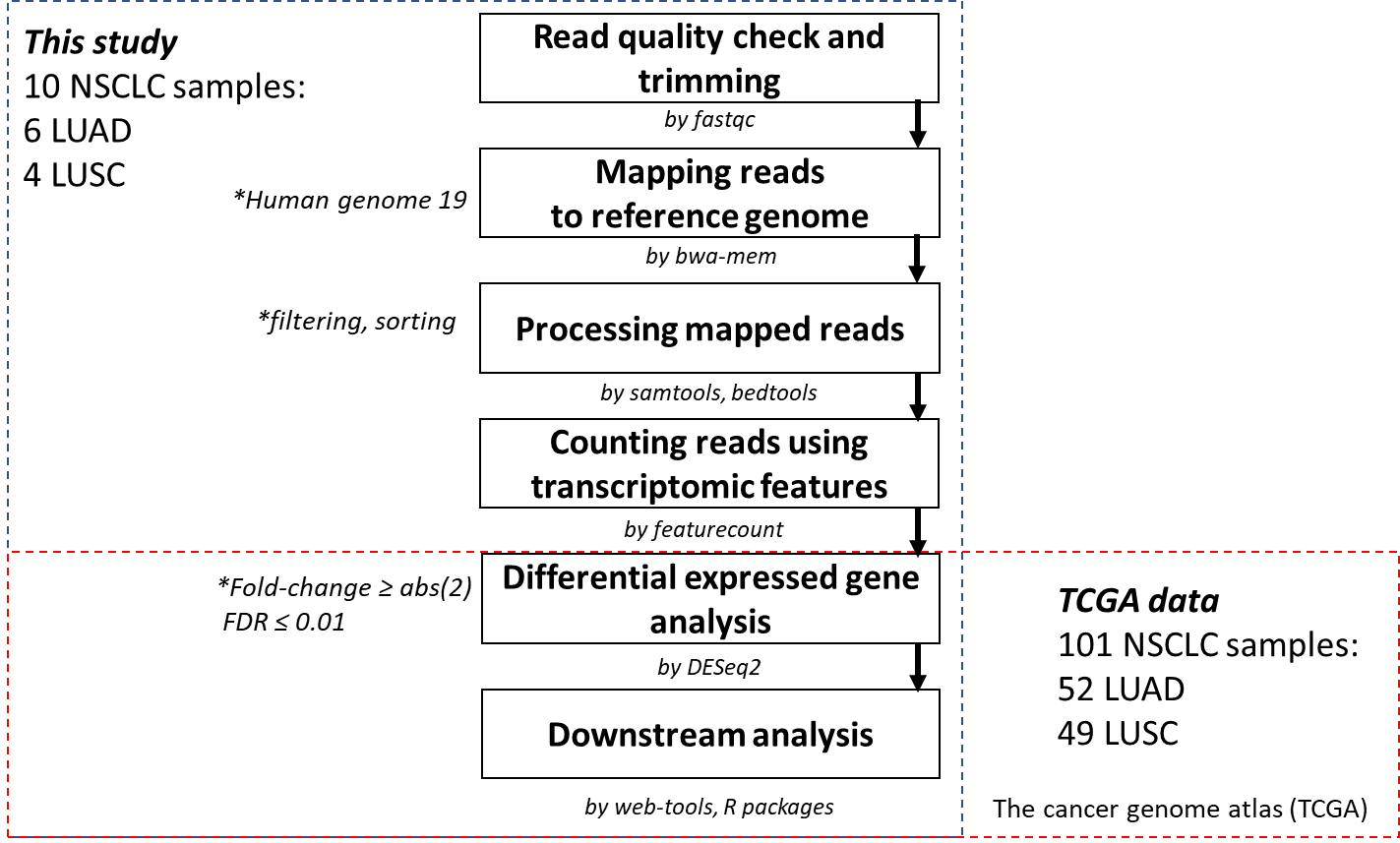 | 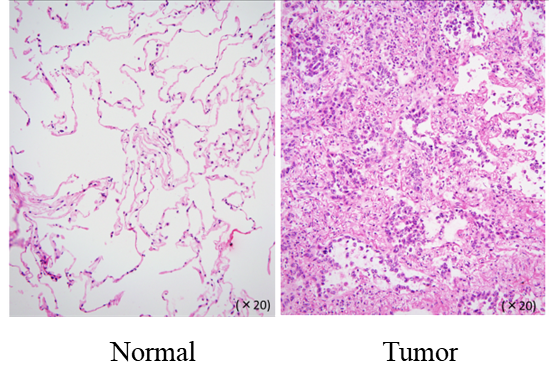 |
| --- | --- |
| **a** | **b** |

**Figure S1.** (**a**) General workflow of RNA-seq analysis using the ten NSCLC cases of this study and re-analysis of 101 NSCLC samples from TCGA for comparison. (**b**) Hematoxylin and eosin (HE) stained slides of lung normal tissue and lung tumor tissue as examples.


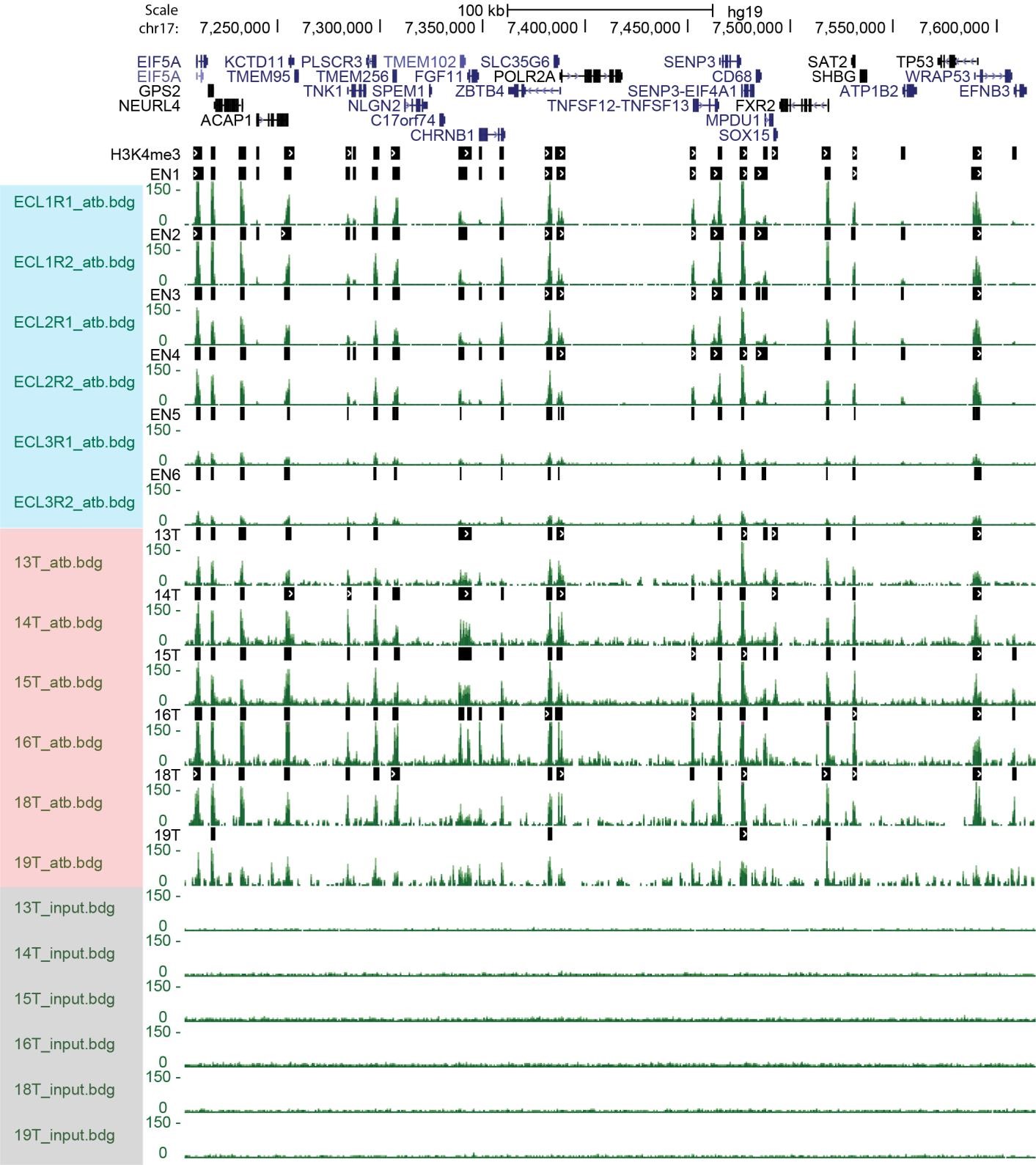


**Figure S2.** A representative UCSC browser view of H3K4me3 ChIP-seq tag density in 6 normal lung tissue samples from ENCODE (blue box, see Methods) and the 6 cases of lung tumor samples in this study (red box) and their input (grey box). H3K4me3 peak profiles of each sample (excluding input samples) is above the corresponding ChIP-seq tracks. The small black rectangles above every track (samples in the blue and red boxes) were the peak calling results from CCAT software, and indicated peak signals of individual samples: EN1, EN2, EN3, EN4, EN5, EN6, 13T, 14T, 15T, 16T, 18T, 19T. Merged peak regions (‘H3K4me3’) were the overlaps of all peak calling signals from (ENCODE) normal and (our study) tumor samples, which are indicated just below the Refseq transcript tracks.


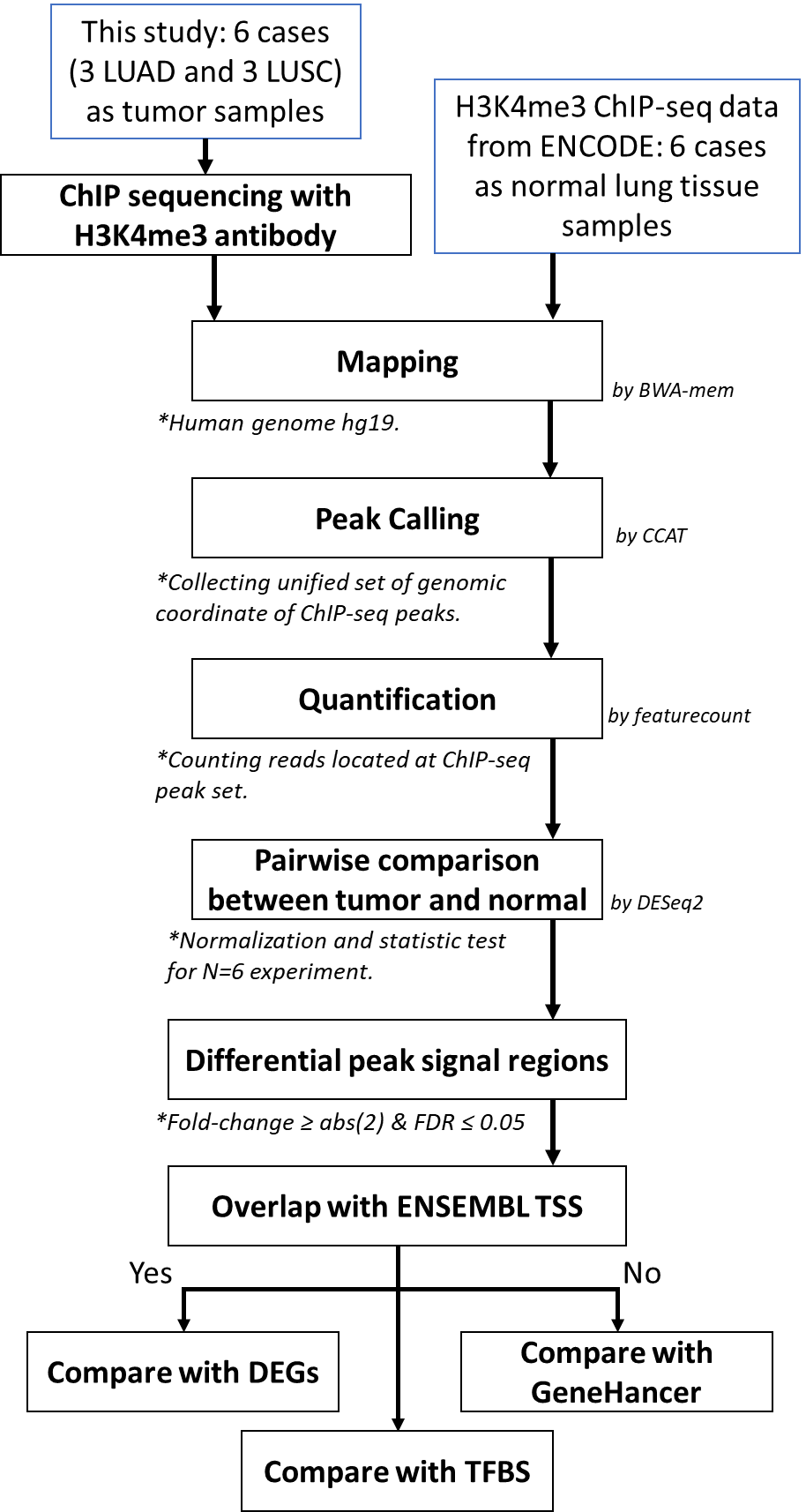


**Figure S3.** General workflow of ChIP-seq analysis.


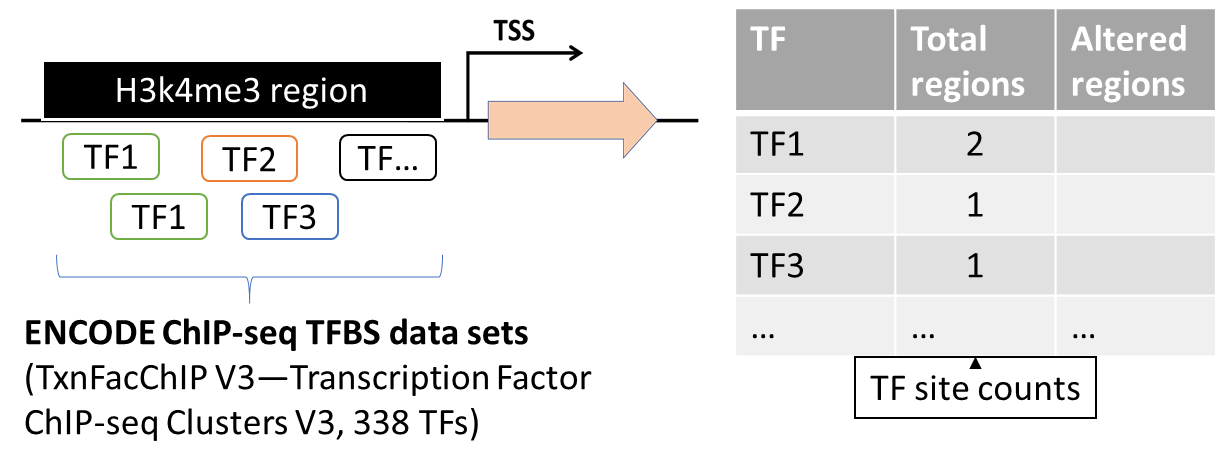

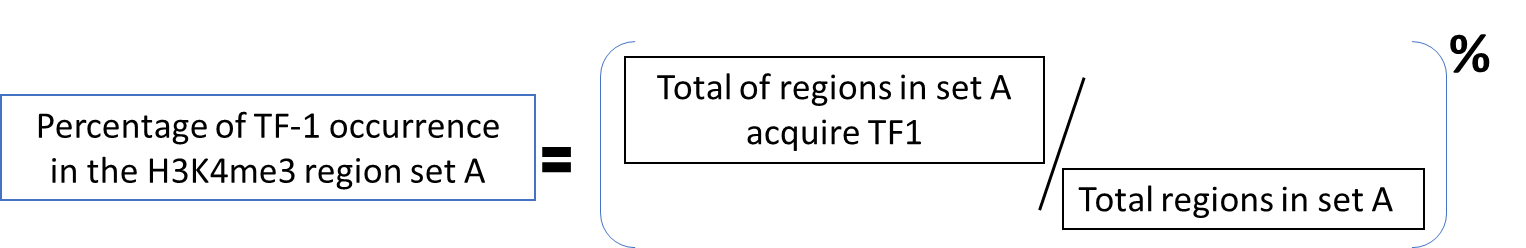

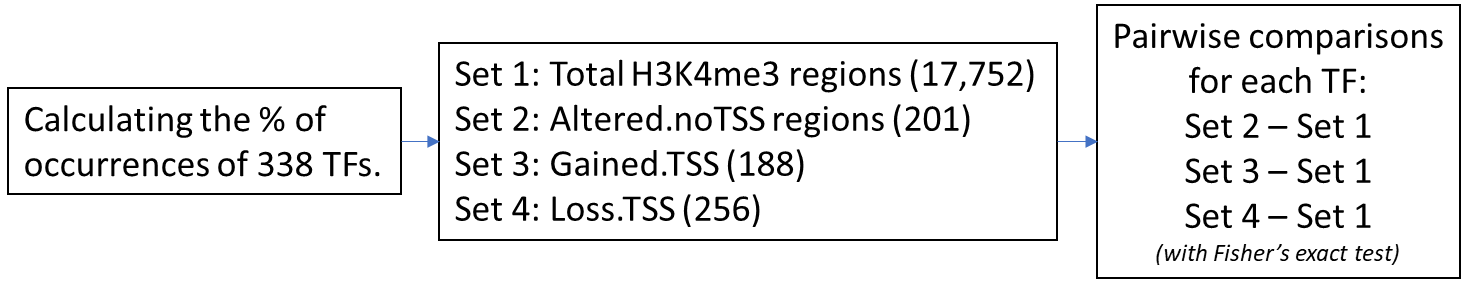


Making plot

**Step 1: Count the number of H3K4me3 regions overlapped with individual TF in each region set.**

**Step 2: Calculate the percentage of occurrence for individual TF in each region set as shown below**

**Step 3: Calculate Fisher’s exact test for individual TF in each pair of region sets as shown below**

**Figure S4.** Process of calculating the percentage of occurrence of individual transcription factors (TFs) in given H3K4me3 region sets.

| 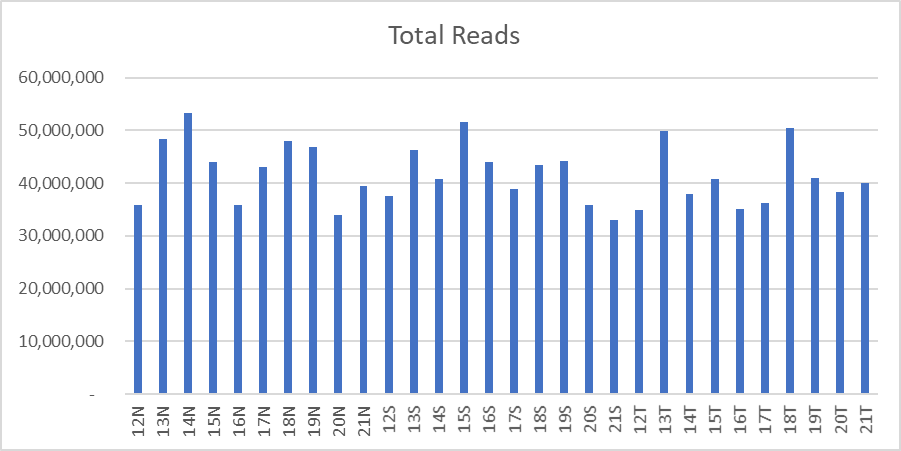 |
| --- |
| **a** |
| 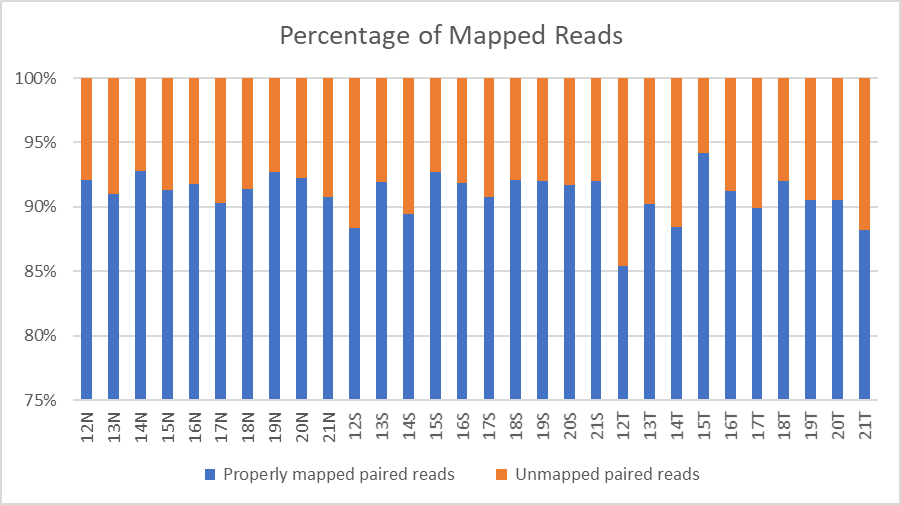 |
| **b** |

**Figure S5.** Total reads (**a**) and percentage of mapped reads (**b**) from RNA-seq analysis of 30 samples from 10 cases (10 normal: ‘N’, 10 stroma: ‘S’, and 10 tumor: ‘T’).


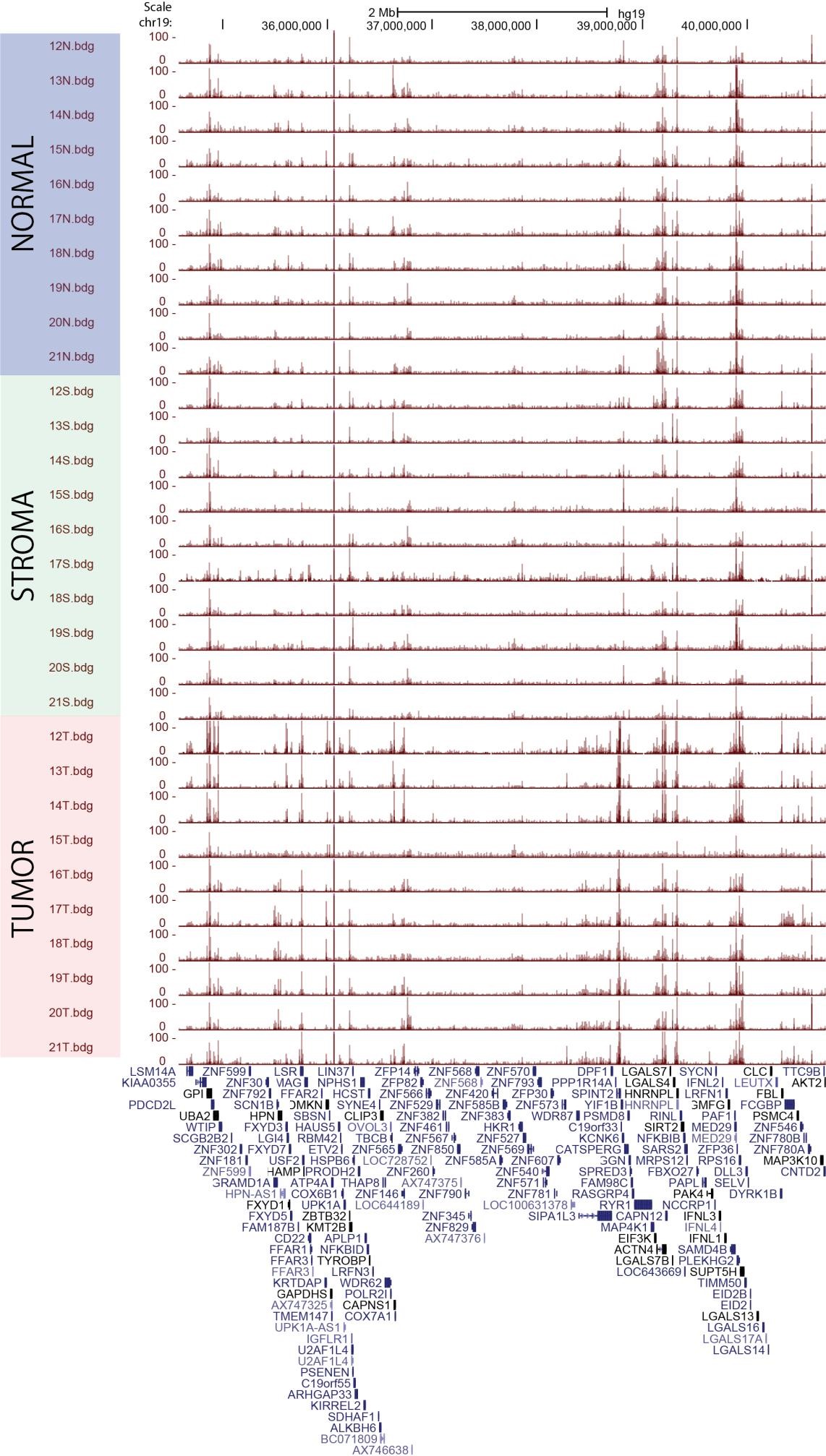


b

**Figure S6.** A representative UCSC browser view of RNA-seq tag density for the 10 lung cancer patient cases. Each case has 3 tracks representing 3 tissue parts: normal (N), stroma (S), and tumor (T).


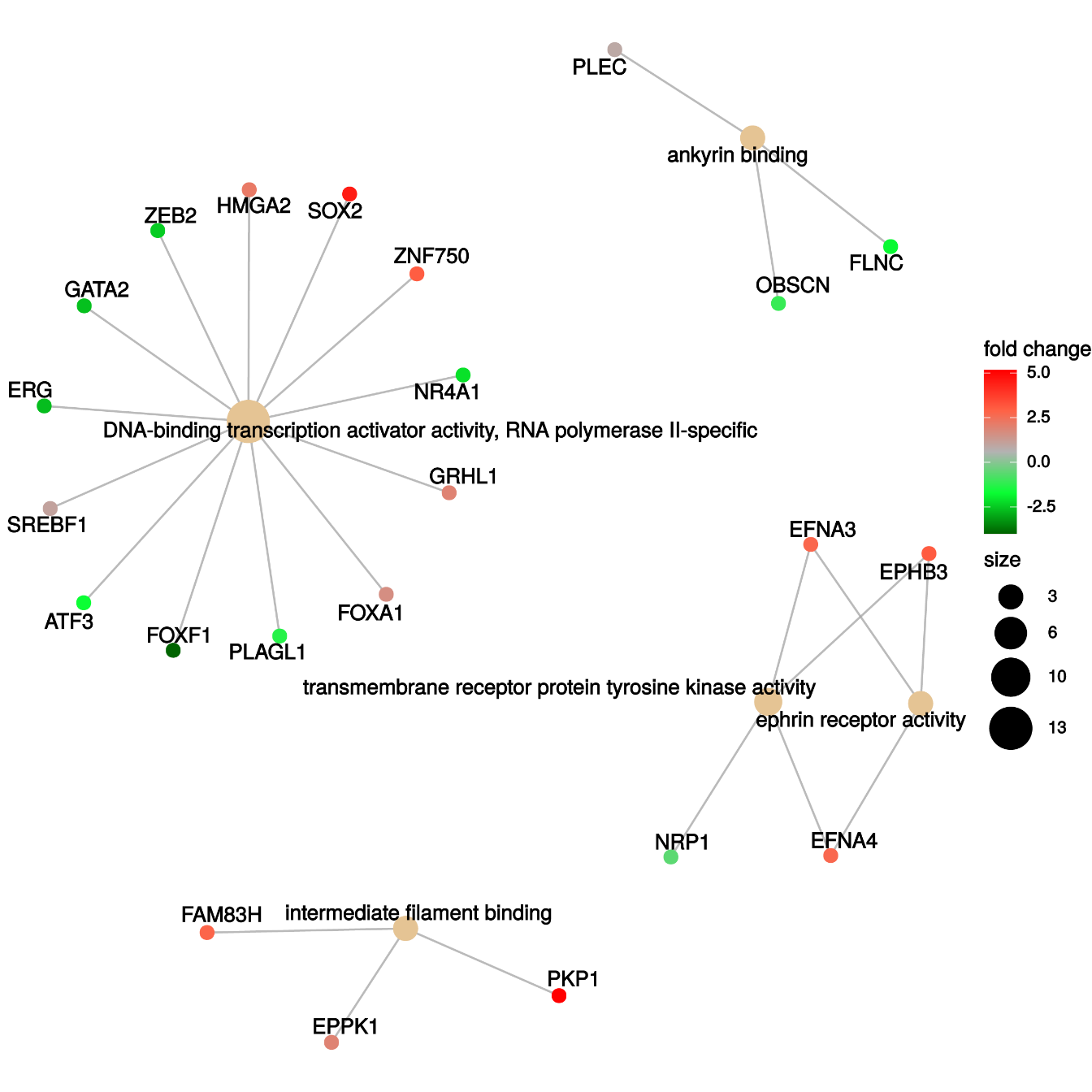


**Figure S7.** The gene ontology (GO) analysis for the 126 DEGs suggested most of the genes were grouped in the same molecular function term: DNA-binding transcription activator activity.

| 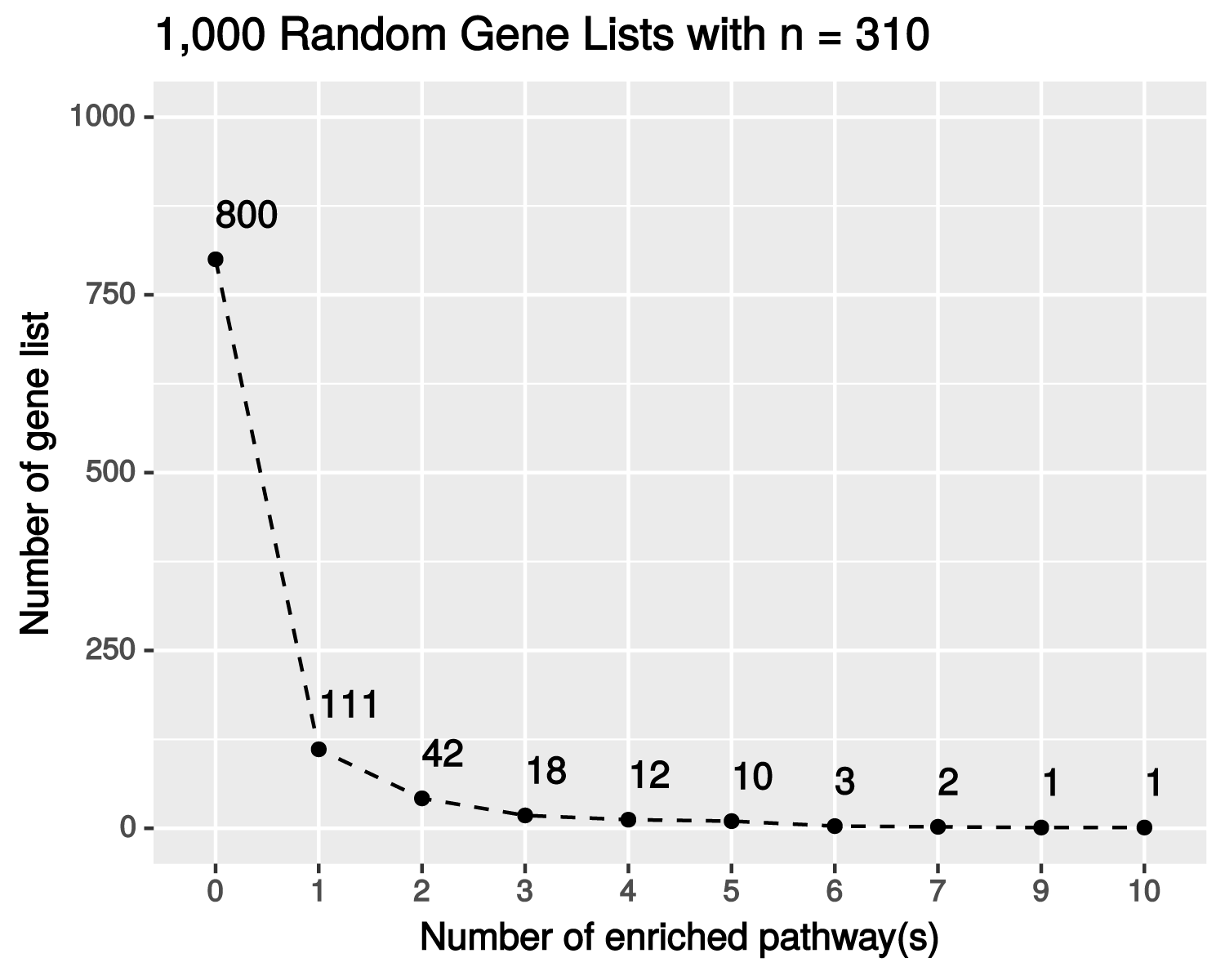 |
| --- |
| **a** |
| 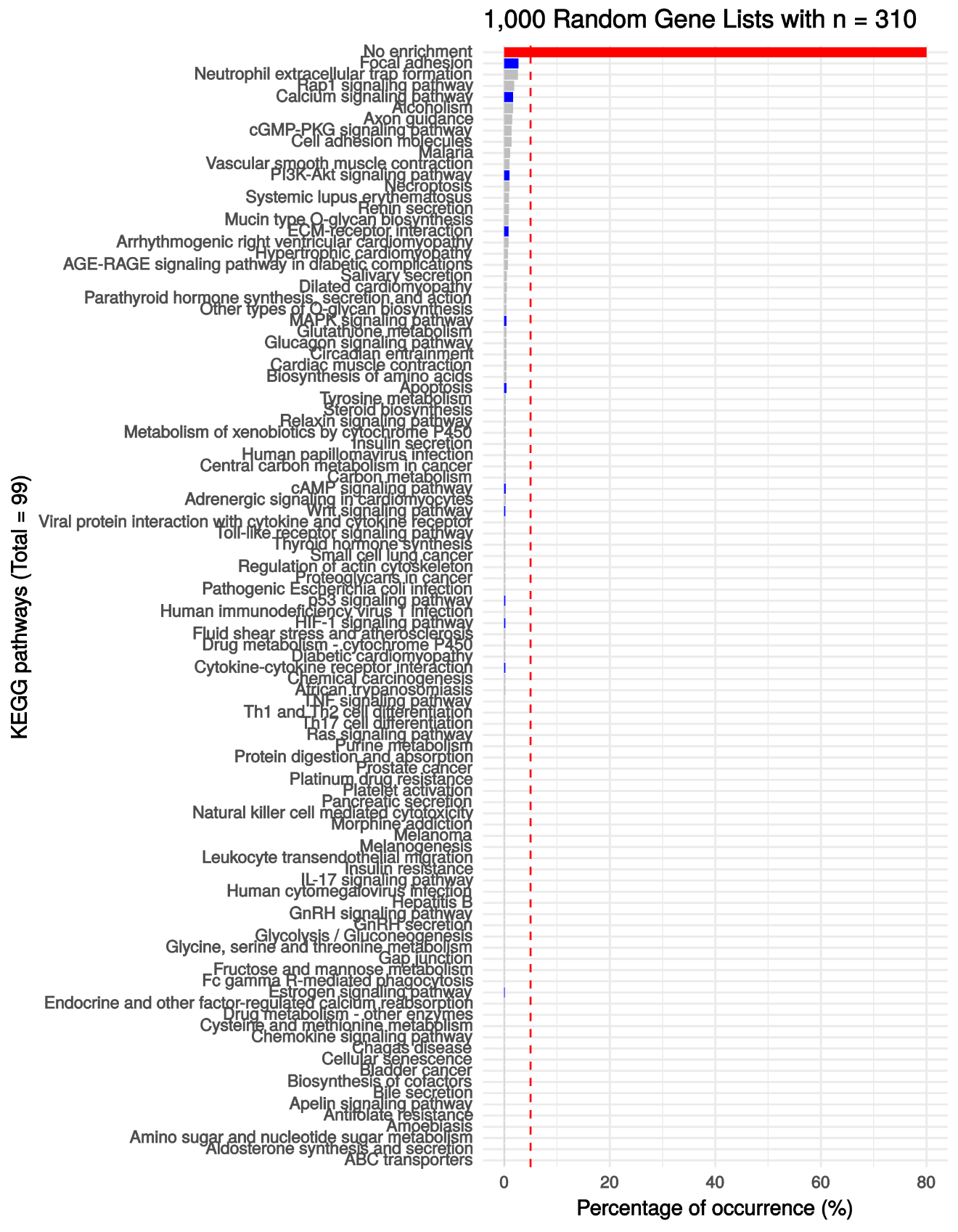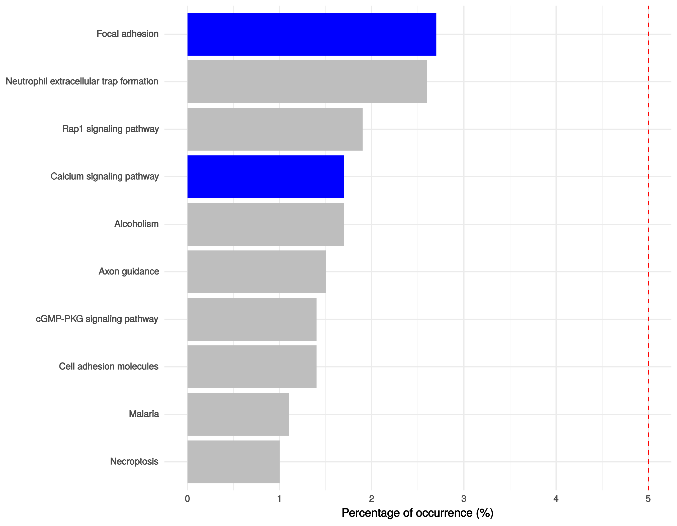 |
| **b** |

**Figure S8.** KEGG pathway enrichment analysis of 1,000 randomly generated gene lists from the 4,118 DEGs that resulted in the RNA-seq analysis. Each gene list consisted of 310 genes, which was the same number of DEGs analyzed in Section 3.5. (a) The chart shows the number of gene lists on the y-axis and the number of pathways enriched on the x-axis. (b) Bar plot shows the percentage of occurrence out of the 1,000 total KEGG pathway results, which are listed on the y-axis. The red bar indicates the highest percentage of occurrence, which was ‘no enrichment’. The blue bars highlights KEGG pathways related to cancer (according to KEGG ‘Pathways in cancer’, hsa05200). The dotted line indicates the 5% percentage of occurrence. Each of the KEGG pathways had less than 5% chance of occurrence in the KEGG enrichment analysis for any of these 1,000 gene lists.

| 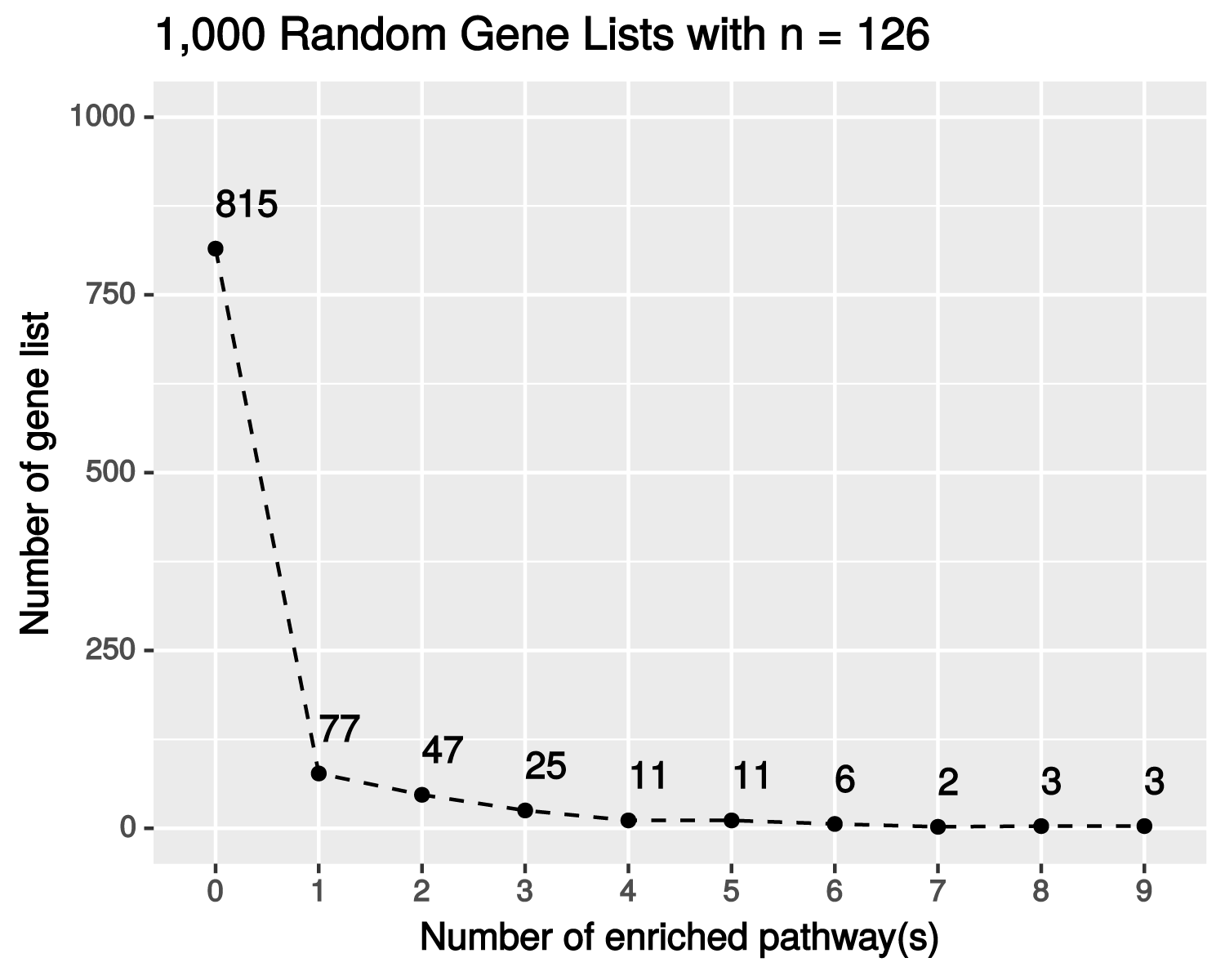 |
| --- |
| **a** |
| 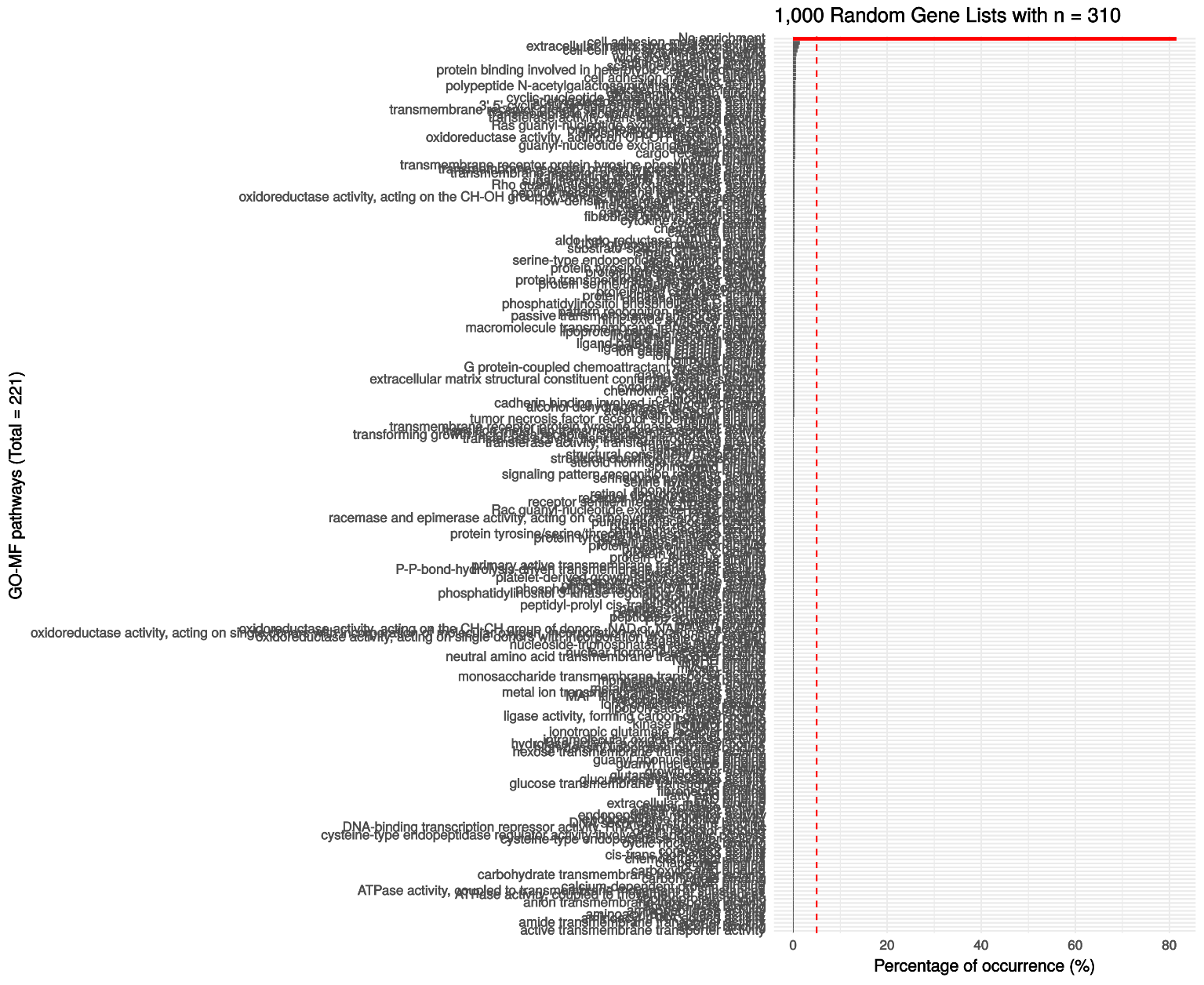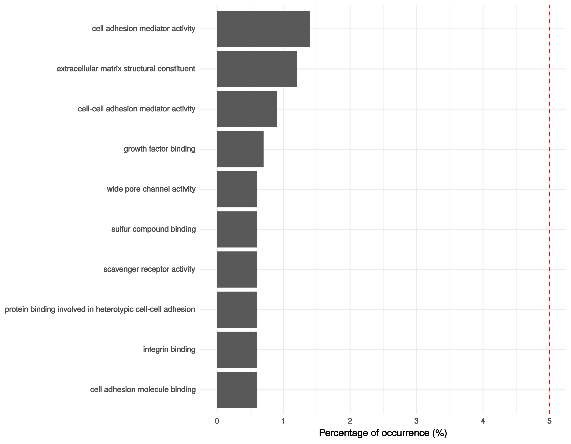 **n = 126** |
| **b** |

**Figure S9.** Gene Ontology (GO) Molecular Function (MF) terms enrichment analysis with of 1,000 randomly generated gene lists from the 4,118 DEGs that resulted in RNA-seq analysis. Each gene list consisted of 126 genes, which was the same number of DEGs analyzed in Section 3.6. (a) The chart shows the number of gene lists in the y-axis and the number of terms enriched on the x-axis. (b) Bar plot shows the percentage of occurrence out of the 1,000 of total GO-MF pathway results, which are listed on the y-axis. The red bar indicates the highest percentage of occurrence, which was ‘no enrichment’. The dotted line indicated the 5% percentage of occurrence. Each of the GO-MF terms had less than 5% chance of occurrence in the GO-MF enrichment analysis for any of these 1,000 gene lists.

**Table S1 (available in the separate excel file).** Patient information.

**Table S2 (available in the separate excel file).** Sources of TCGA samples.

**Table S3 (available in the separate excel file).** Sources of adult normal lung tissue ChIP-Seq data from ENCODE.

**Table S4 (available in the separate excel file).** RNA-Seq mapping quality.

**Table S5 (available in the separate excel file).** Total of 4118 DEGs from RNA-Seq analysis.

**Table S6 (available in the separate excel file).** Top 15 up-/downregulated genes in LUAD and LUSC.

**Table S7 (available in the separate excel file).** KEGG pathway analysis results from DEGs (tumor versus normal) of LUAD and LUSC.

**Table S8 (available in the separate excel file).** ChIP-SSeq mapping quality.

**Table S9 (available in the separate excel file).** Total of 645 tumor-altered H3K4me3 from ChIP-Seq analysis.

**Table S10 (available in the separate excel file).** Altered H3K4me3 regions overlap with DEGs.

**Table S11 (available in the separate excel file).** Gene Ontology (GO) analysis and list of DEGs associated with somatically altered proximal H3K4me3.

**Table S12 (available in the separate excel file).** Predicted GeneHancer regions with targeted genes.

**Table S13 (available in the separate excel file).** Gene Ontology (GO) analysis and list of DEGs associated with potential H3K4me3-marked enhancers.

**Table S14 (available in the separate excel file).** Percentage of TFs over-represented in H3K4me3 regions.

**Publisher’s Note:** MDPI stays neutral with regard to jurisdictional claims in published maps and institutional affiliations.

| 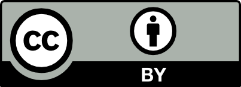 | © 2021 by the authors. Licensee MDPI, Basel, Switzerland. This article is an open access article distributed under the terms and conditions of the Creative Commons Attribution (CC BY) license (http://creativecommons.org/licenses/by/4.0/). |
| --- | --- |
